# Supplementary figures and images for: A quantitative in vivo CRISPR-imaging platform identifies regulators of hyperplastic and hypertrophic adipose morphology in zebrafish
Source: eLife. 2026 Apr 22;14:RP107327. doi: 10.7554/eLife.107327 (PMC13102392; doi:10.7554/eLife.107327)

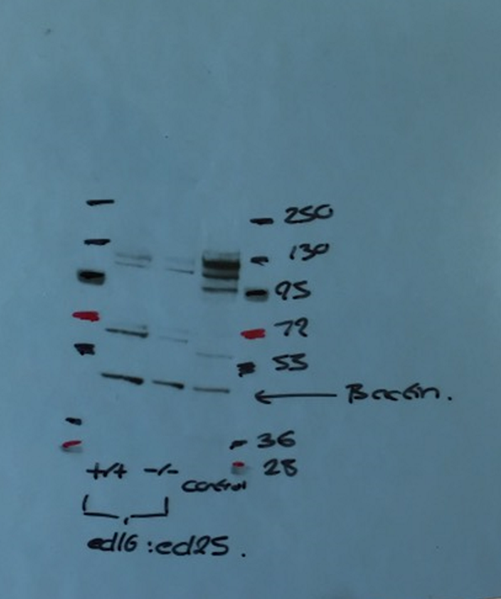

Supplement: Figure 6—source data 1. — Note, ‘ed16’ and ‘ed25’ in the blot correspond to the foxp1aed116 and foxp1bed125 alleles in the manuscript. The red box highlights the section of the blot shown in Figure 6c. [file elife-107327-fig6-data1.zip › Figure6C_source_data/Figure 6C-source data 1.tif]

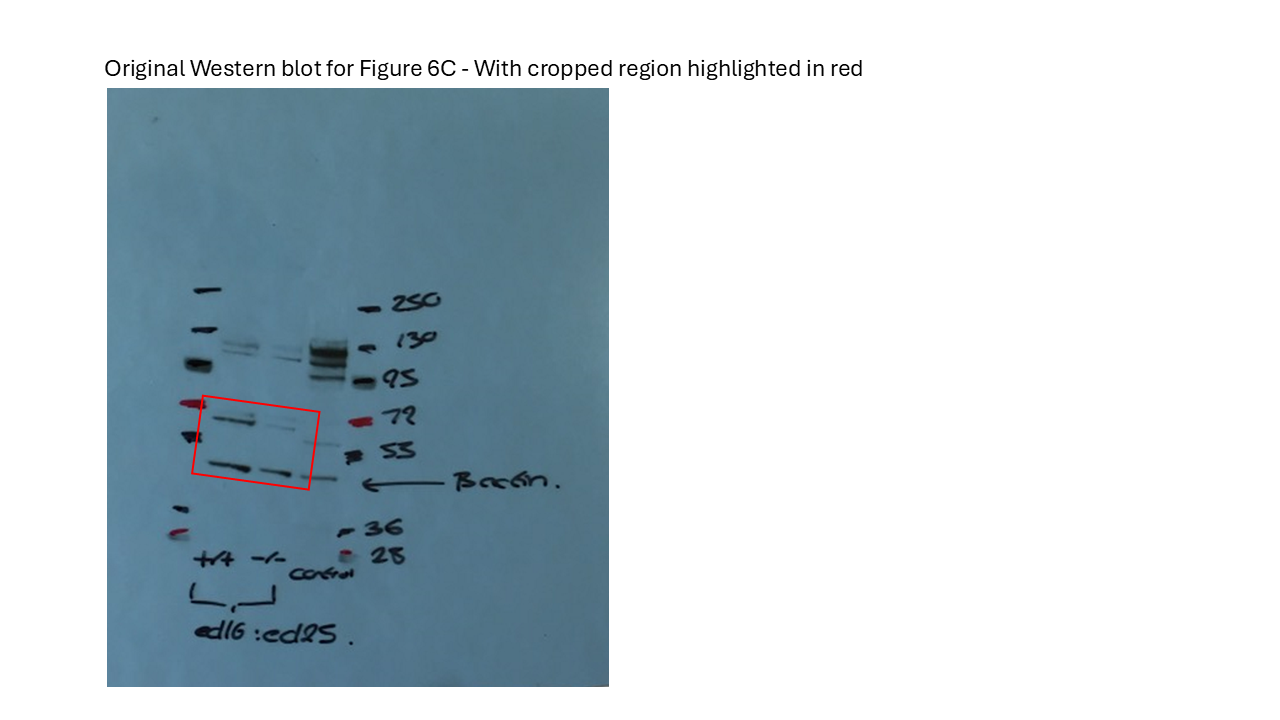

Supplement: Figure 6—source data 2. [file elife-107327-fig6-data2.zip › Figure6C_source_data_indicating_bands/Figure6C_source_data_indicating_bands.tif]

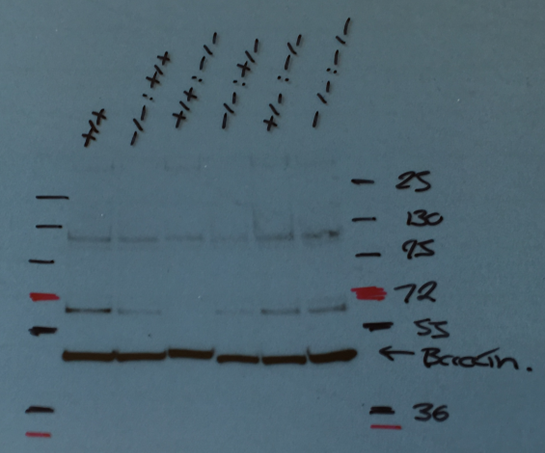

Supplement: Figure 6—figure supplement 1—source data 1. — Note, ‘ed16’ in the blot corresponds to the foxp1aed116 allele in the manuscript. ‘ed23’ in the blot corresponds to an allele not included and not relevant to this article. The red box highlights the section of the blot shown in Figure 6—figure supplement 1c. [file elife-107327-fig6-figsupp1-data1.zip › 150312_0_data_set_3783870_tcrpk8/Figure 6 – Figure Supplement 1-source_data/Figure 6 - Figure Supplement 1F-source_data.tif]

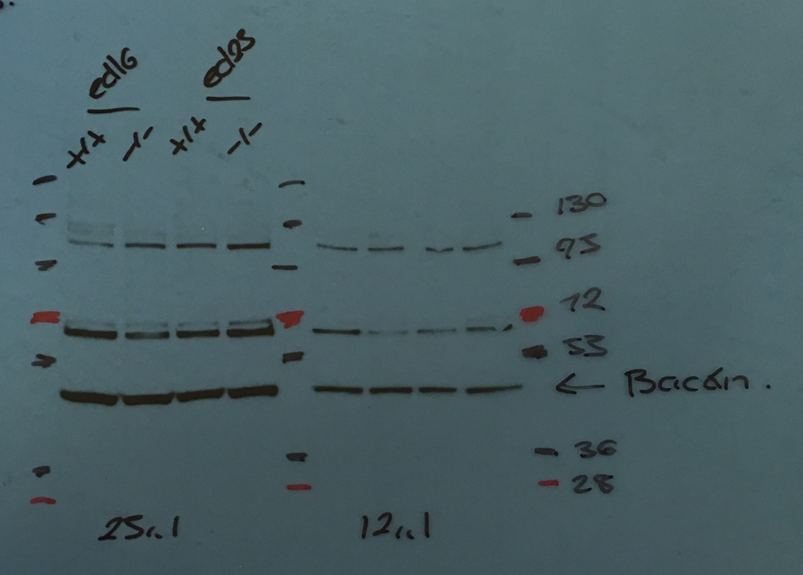

Supplement: Figure 6—figure supplement 1—source data 1. — Note, ‘ed16’ in the blot corresponds to the foxp1aed116 allele in the manuscript. ‘ed23’ in the blot corresponds to an allele not included and not relevant to this article. The red box highlights the section of the blot shown in Figure 6—figure supplement 1c. [file elife-107327-fig6-figsupp1-data1.zip › 150312_0_data_set_3783870_tcrpk8/Figure 6 – Figure Supplement 1-source_data/Figure 6 – Figure Supplement 1C-source_data.tif]

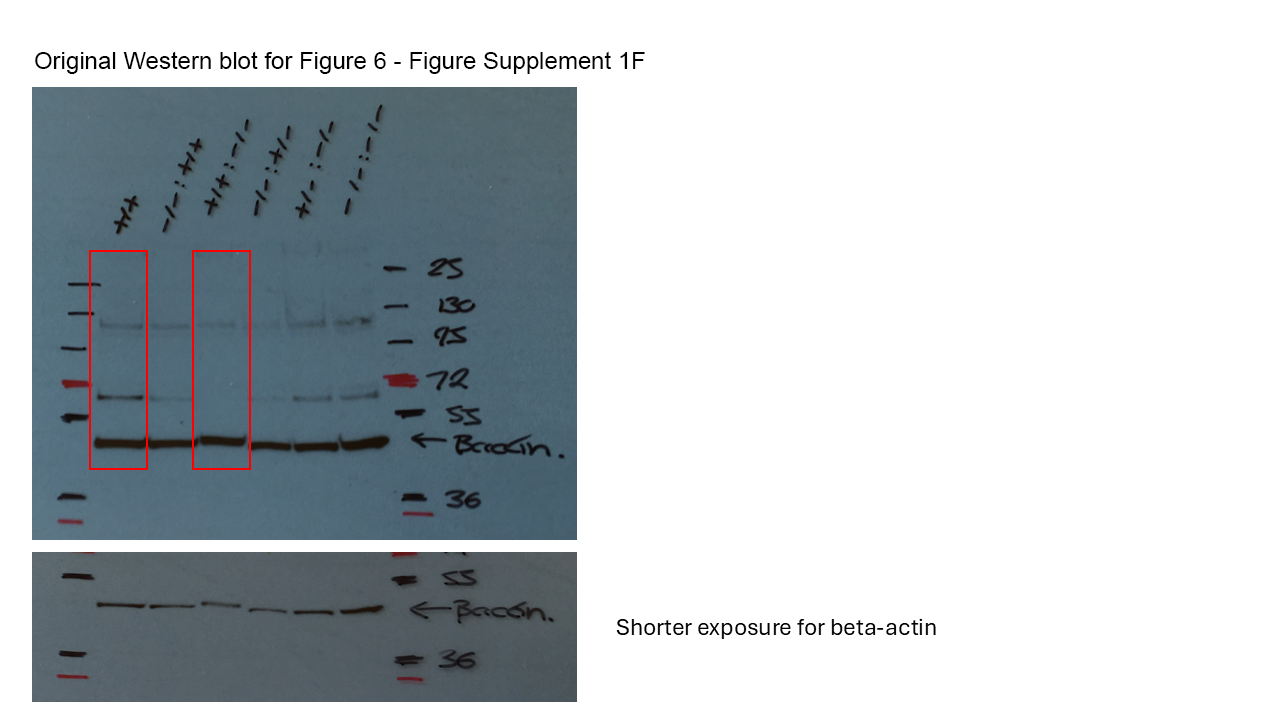

Supplement: Figure 6—figure supplement 1—source data 2. — Note, these are mutants for foxp1bed125 and an additional foxp1a allele not included in this article. In the blot, genotypes are separated by a colon with the foxp1a allele first, and foxp1bed125 second (e.g. foxp1a allele;ed125). So, +/+; –/– corresponds to a foxp1a wild-type and foxp1bed125 homozygous mutant genotype. The red boxes highlight the lanes shown in Figure 6—figure supplement 1f. [file elife-107327-fig6-figsupp1-data2.zip › 150312_0_data_set_3783871_tcrpk8/Figure 6 – Figure Supplement 1-source_data_bands_indicated/Figure 6 - Figure Supplement 1F-source_data_bands_indicated.tif]

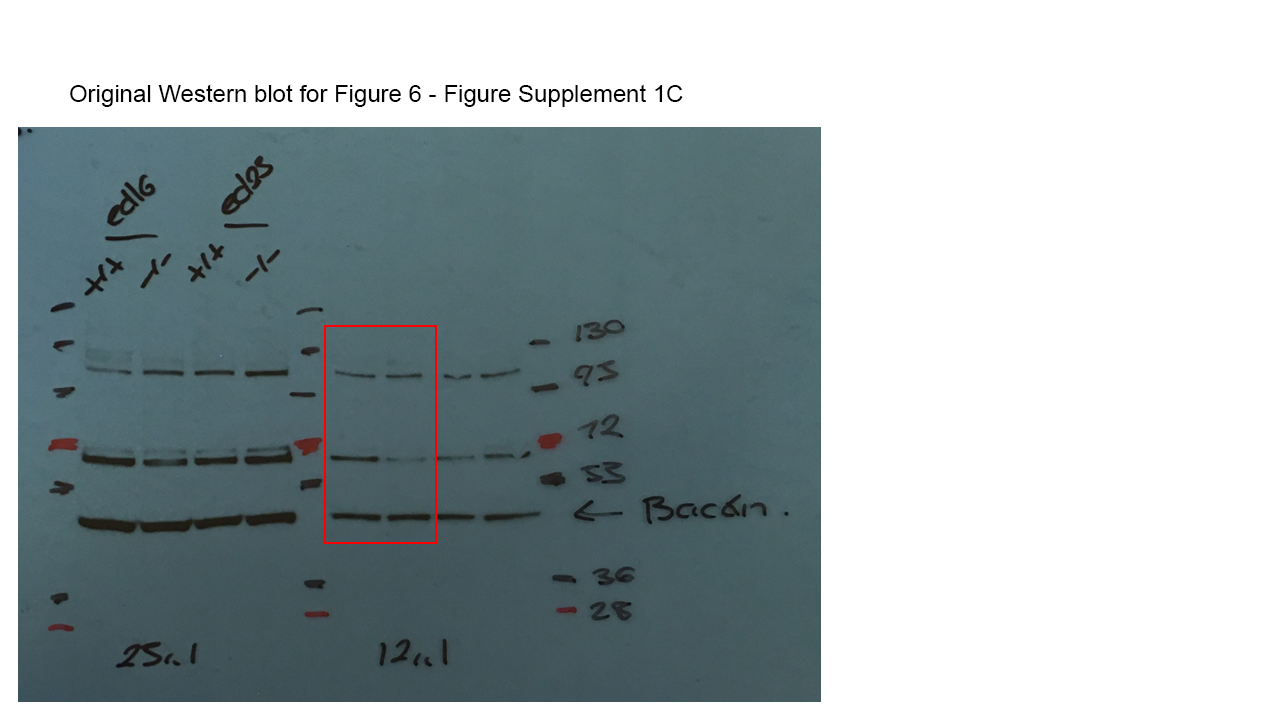

Supplement: Figure 6—figure supplement 1—source data 2. — Note, these are mutants for foxp1bed125 and an additional foxp1a allele not included in this article. In the blot, genotypes are separated by a colon with the foxp1a allele first, and foxp1bed125 second (e.g. foxp1a allele;ed125). So, +/+; –/– corresponds to a foxp1a wild-type and foxp1bed125 homozygous mutant genotype. The red boxes highlight the lanes shown in Figure 6—figure supplement 1f. [file elife-107327-fig6-figsupp1-data2.zip › 150312_0_data_set_3783871_tcrpk8/Figure 6 – Figure Supplement 1-source_data_bands_indicated/Figure 6 – Figure Supplement 1C_source_data_bands_indicated.tif]
